# Dengue Infection - Recent Advances in Disease Pathogenesis in the Era of COVID-19

**DOI:** 10.3389/fimmu.2022.889196

**Published:** 2022-07-06

**Authors:** Yean Kong Yong, Won Fen Wong, Ramachandran Vignesh, Indranil Chattopadhyay, Vijayakumar Velu, Hong Yien Tan, Ying Zhang, Marie Larsson, Esaki M. Shankar

**Affiliations:** ^1^Laboratory Centre, Xiamen University Malaysia, Sepang, Malaysia; ^2^Department of Medical Microbiology, Faculty Medicine, University of Malaya, Kuala Lumpur, Malaysia; ^3^Preclinical Department, Royal College of Medicine Perak (UniKL RCMP), Universiti Kuala Lumpur, Ipoh, Malaysia; ^4^Cancer and Microbiome Biology, Department of Life Sciences, Central University of Tamil Nadu, Thiruvarur, India; ^5^Division of Microbiology and Immunology, Emory Vaccine Center, Yerkes National Primate Research Center, Emory University, Atlanta, GA, United States; ^6^Department of Pathology and Laboratory Medicine, Emory National Primate Research Center, Emory University, Atlanta GA, United States; ^7^School of Traditional Chinese Medicine, Xiamen University Malaysia, Sepang, Malaysia; ^8^Chemical Engineering, Xiamen University Malaysia, Sepang, Malaysia; ^9^Molecular Medicine and Virology, Department of Biomedical and Clinical Sciences, Linköping University, Linköping, Sweden; ^10^Infection Biology, Department of Life Sciences, Central University of Tamil Nadu, Thiruvarur, India

**Keywords:** dengue (DENV), pathogenesis, plasmablasts, inflammasome, cytokine storm, endothelial dysfunction, antibody-dependent enhancement (ADE), COVID-19

## Abstract

The dynamics of host-virus interactions, and impairment of the host’s immune surveillance by dengue virus (DENV) serotypes largely remain ambiguous. Several experimental and preclinical studies have demonstrated how the virus brings about severe disease by activating immune cells and other key elements of the inflammatory cascade. Plasmablasts are activated during primary and secondary infections, and play a determinative role in severe dengue. The cross-reactivity of DENV immune responses with other flaviviruses can have implications both for cross-protection and severity of disease. The consequences of a cross-reactivity between DENV and anti-SARS-CoV-2 responses are highly relevant in endemic areas. Here, we review the latest progress in the understanding of dengue immunopathogenesis and provide suggestions to the development of target strategies against dengue.

## Introduction

Dengue is an infectious disease transmitted between humans by Aedes mosquitoes, especially across the tropical and subtropical latitudes afflicting ~400 million people annually, of which 100 million manifests clinically (1). Estimates by the World Health Organization (WHO) suggest that the global consequence of dengue is exponentially increasing and almost half of the global population is at risk for contracting the infection (2). Dengue is caused by at least four different dengue virus (DENV) serotypes, DENV1, DENV2, DENV3, and DENV4. In recent years, most endemic countries, e.g., Asia-Pacific and Latin American nations, are reporting almost all the four different DENV serotypes (3), which altogether cause ~20000 deaths annually (4). The surge in endemicity is attributed to rapid urbanization, increasing population density and a rise in vector-breeding sites (5). *Aedes aegypti* (*A. aegypti*) represents the major vector that transmits dengue in urban areas, whereas the density of *A. albopictus*, the secondary vector, is dramatically expanding globally (6, 7). Given the context of global warming, the environment appears to be appropriate for the breeding of *Aedes* mosquitos, that in turn, would drive the dissemination of the dengue disease further (8).

DENV is a member of the *Flavivirus* genus of the *Flaviviridae* family. DENV has a spherical shape with icosahedral symmetry. It is a single-stranded positive sense RNA virus with a genome size of ~11 kb (9). It has a single long open reading frame (ORF) that encodes for three structural and seven non-structural (NS) proteins. The structural proteins are capsid (C), pre-membrane/membrane (prM/M), and envelope glycoproteins (E), and the NS proteins are NS1, NS2A, NS2B, NS3, NS4A, NS4B, and NS5. The NS proteins are not present in the virion, but contribute to viral replication and immune evasion within an infected cell (10–12). Of all the NS proteins, only NS1 is displayed on the infected cell surfaces, and is eventually secreted into the systemic circulation making it an appropriate diagnostic marker. While detection of DENV genomic RNA (by RT-PCR) and NS1 are the mainstay of laboratory diagnosis, detection of NS1 has an edge over the detection of DENV genomic RNA. Albeit being highly sensitive, the RT-PCR viral detection rate has a finer window period where detection rate appears to drop dramatically by day 4 onwards following the onset of clinical symptoms (13, 14). Conversely, NS1 can be detected in the serum for a wider time range, viz., from the first day of symptom onset, with the concentration average of 2 µg/ml (can reach as high as 50 µg/ml in the same cases) (15) that remains detectable between 9 and 18 days (16, 17). Furthermore, the level of NS1 appears to correlate with disease severity, rendering it an ideal biomarker, both for diagnosis as well as prognosis in dengue (12, 18, 19).

Dengue infection results in clinical manifestations ranging from a predominantly asymptomatic or a symptomatic, mild undifferentiated febrile illness to severe life-threatening dengue hemorrhagic fever (DHF) and dengue shock syndrome (DSS) that can be fatal (6). The hallmarks of severe dengue are coagulopathy and leaky vasculature that eventually can lead to life-threatening hemodynamic shock and organ failure (20, 21). Evidence suggests that young age, female biological sex, high body-mass index, virus strain, and genetic variants of the human MHC class I–related sequence B, and phospholipase C epsilon 1 genes could serve as risk factors for development of severe dengue (6).

The human and economic burden caused by dengue fever remains enormous as specific antiviral drugs, or effective vector-control mechanisms is lacking. Although no specific treatment is available, prompt hospital admission, triage, and fluid restoration are critical to prevent death (22). In 2016, (CYD-TDV) DengVaxia^®^, a tetravalent vaccine was licensed to prevent severe secondary dengue in seropositive individuals. However, the vaccine was not recommended for seronegative individuals as the levels of vaccine-induced antibodies reportedly decreased over time (23). The current review will focus on some of the hitherto poorly understood disease immunopathogenesis mechanisms as well as potential interventions against DENV infection.

## Dengue Immunopathogenesis: A Brief Overview

The onset of severe dengue often occurs during the defervescence stage after peak viremia suggesting that the host immune responses are implicated in viral clearance (6, 24), inferring that life-threatening dengue involves a complex interplay between virus and the host (25). Natural infection with one of the serotypes confers long-lasting immunity to subsequent infection with the same serotype. However, subsequent infection with heterotypic serotypes often results in severe immunopathological manifestations, triggered early during the course of disease (26). This, at least in part, could be attributed to a phenomenon known as *original antigenic sin* that engenders ineffective T and B cell responses and potentially harmful manifestations, particularly during secondary infection. The complex interplay between these factors may eventually lead to both antibody-dependent enhancement (ADE), antibody-dependent cellular cytotoxicity (ADCC), cytokine storm (hypercytokinemia), aberrant activation of the complement system (CS), as well as endothelial dysfunction, culminating in severe clinical dengue (27, 28).

## Original Antigenic Sin and Antibody-Dependent Enhancement

**Figure 1:**
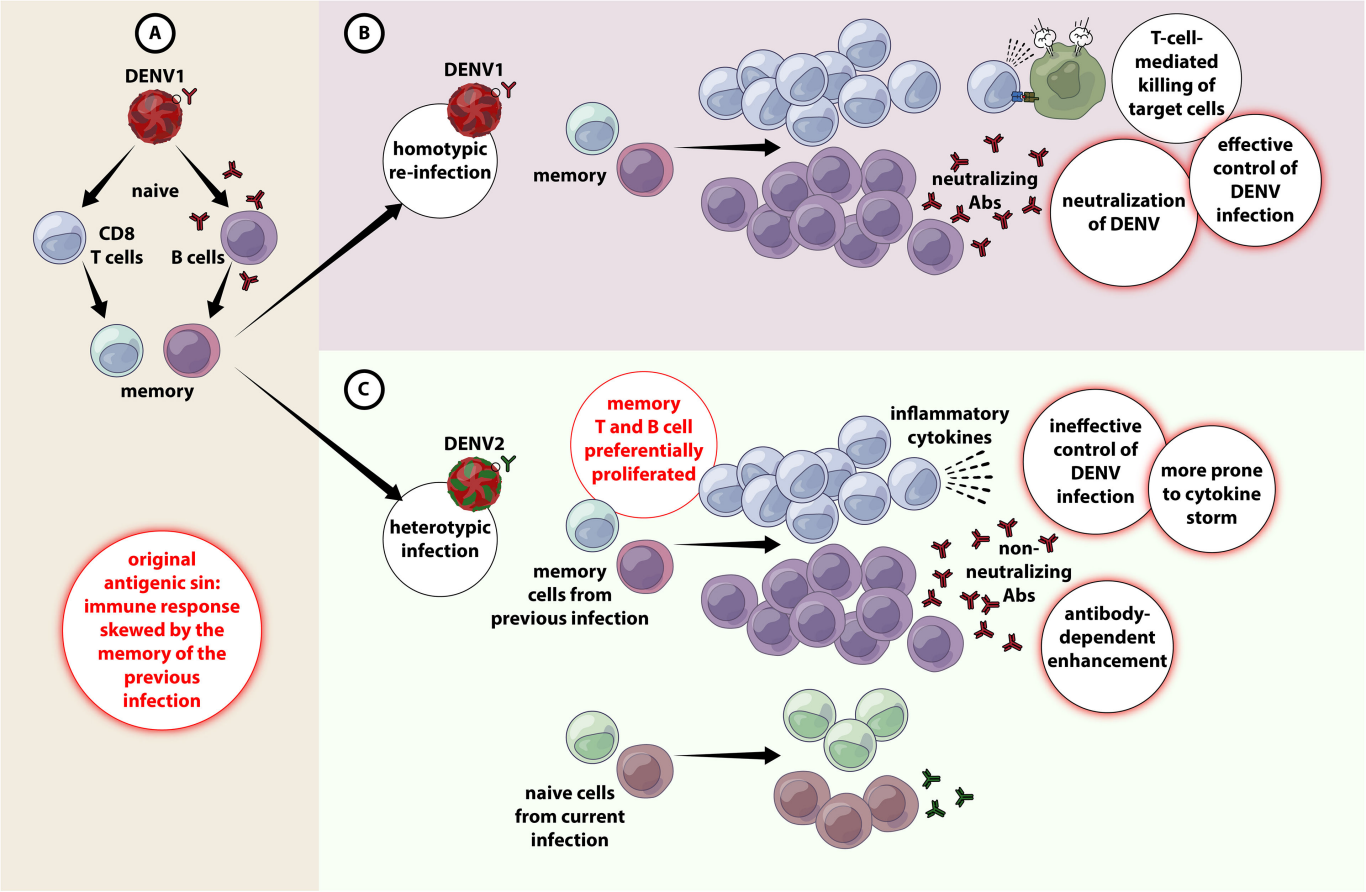
Original antigenic sin and antibody-dependent enhancement in DENV infection. **(A)** When primary infection occurs with e,g. DENV1, resulting in activation of adaptive immune responses (both T and B cells) DENV1-specific T cells are selected, activated, and clonally expanded to combat infection. Upon termination of primary infection, memory DENV1–specific T cells and B cells are formed and are retained with higher frequency compared to other naïve cells. **(B)** A secondary infection with the same serotype of DENV (e.g. DENV1) for the second time (homotypic infection), the virus will evoke a memory response that entails in the effective containment of DENV1 by highly specific T and B cell responses. **(C)** A secondary challenge with a different serotype of DENV (e.g. DENV2) (heterotypic infection), there is a chance that the cross-reactive memory T and B cells get preferentially activated, proliferated over the DENV2-specific T and B cells. The cross-reactive DENV1–specific adaptive immune responses outcompete naïve T cells that would be more specific for DENV2, resulting in an expanded memory T cell pool that is of low specificity for DENV2 and poor viral clearance. Antibody-dependent enhanced replication also has the potential to occur during a secondary, heterologous infection.

Although both T and B cell responses play a paramount role in combating DENV infection (29), they could be pathological during secondary infection due to *original antigenic sin*. Because the four DENV serotypes share ~80% homology in amino acid sequences, cross-reactivity is common (30). Hence, during a heterotypic infection, the preexisting memory T and B cells rapidly become activated to proliferate to enter into the effector phase (26). As protective adaptive immunity is more efficient against homotypic than heterotypic reinfection (31), seeing that cross-reactive responses may have suboptimal avidity and affinity towards the epitopes of the secondary-infecting virus (27). These cross-reactive T cells often exhibit lower cytotoxicity yet secreting higher abundance of several pro-inflammatory cytokines (32), rendering viral control ineffective as well as exaggerated release of pro-inflammatory cytokines leading to cytokine storm and endothelial dysfunction (26, 33) (**Figure 1**).

Akin to T cells, the titer of DENV-specific antibodies produced from prior infection increases substantially during secondary dengue, and they are predominantly non-neutralizing. Binding of these cross-reactive non-neutralizing antibodies with DENV virions could set in motion both extrinsic and intrinsic forms of ADE. Extrinsic ADE occurs when non-neutralizing antibodies forming a virus-antibody complex are recognized and engulfed by other uninfected cells, e.g., monocytes, macrophages, dendritic cells (DCs) and mast cells, *via* their gamma Fc receptors (FcγR), particularly FcγRI (CD64) and FcγRII (CD32), resulting in an increase in the frequency of DENV-infected cells, and subsequent upsurge in viral production (28, 34). Intrinsic ADE on the other hand, was first observed in Ross River virus (RRV) where incubation of RRV anti-RRV IgG had resulted in ADE-mediated persistent productive infection of macrophages for extended time periods. Further investigations showed that the entry of virus *via* Fcγ-antibody complexes will bypass TLR3 and TLR7 signaling leading to a Th2-biased immune responses and increased viral production (35, 36). Later the same phenomena was also observed in DENV (37, 38), where viral entry *via* FcγR often produces <10-fold virions than through host receptors (e.g mannose receptor; CD205), CD14-associated protein, heat shock protein (HSP79/HSP90), DC-SIGN (CD209)) (39, 40). While intrinsic ADE increases the quantity of new infectious virions produced by an infected cell, extrinsic ADE increases the mass of cells infected by DENV, both contributing to enhanced viral replication and disease severity (41).

Apart from enhanced viral replication, the FcγR-bearing immune cells also become hyperactivated, leading to augmented production of cytokines and vasoactive mediators entailing in cytokine storm (hypercytokinemia) and endothelial dysfunction (25, 42, 43). The presence of reactive, non-neutralizing IgG is the greatest risk factor for development of DSS. These antibody isotypes have enhanced affinity for the FcγRIIIA (CD16) due to a-fucosylated FC glycans, which results in rapid reduction of platelets leading to significant thrombocytopenia (44, 45). Recently, B cells has been shown to be directly susceptible to DENV infection. The infected B cells can support low viral replication *via* CD300a (46). Interestingly, direct DENV infection of B cell induces their proliferation *in vivo* and formation of plasmablasts *in vitro* (46). Though ADE is not observed in B cells, the production of massive amounts of non-neutralizing antibodies during secondary infection likely has an association with disease severity (46).

## Interaction of the Complement System With DENV

The complement system composed of ~50 plasma proteins is pivotal to first-line immune surveillance and are found in inactive forms in the circulation. The proteins can be activated *via* three pathways, i.e. the classical, (binding of C1q with antigen-antibody complex), lectin (binding by mannose binding lectins (MBLs) and the alternate (via spontaneous activation of C3 protein) pathways. Once activated, the complement proteolytic cascade will initiate the expression of chemokines, recruitment of monocytes and granulocytes *via* anaphylatoxins C3a and C5a, opsonization, release of pro-inflammatory cytokines, and direct killing of infected cells by deposition of membrane attack complex (MAC).

**Figure 2:**
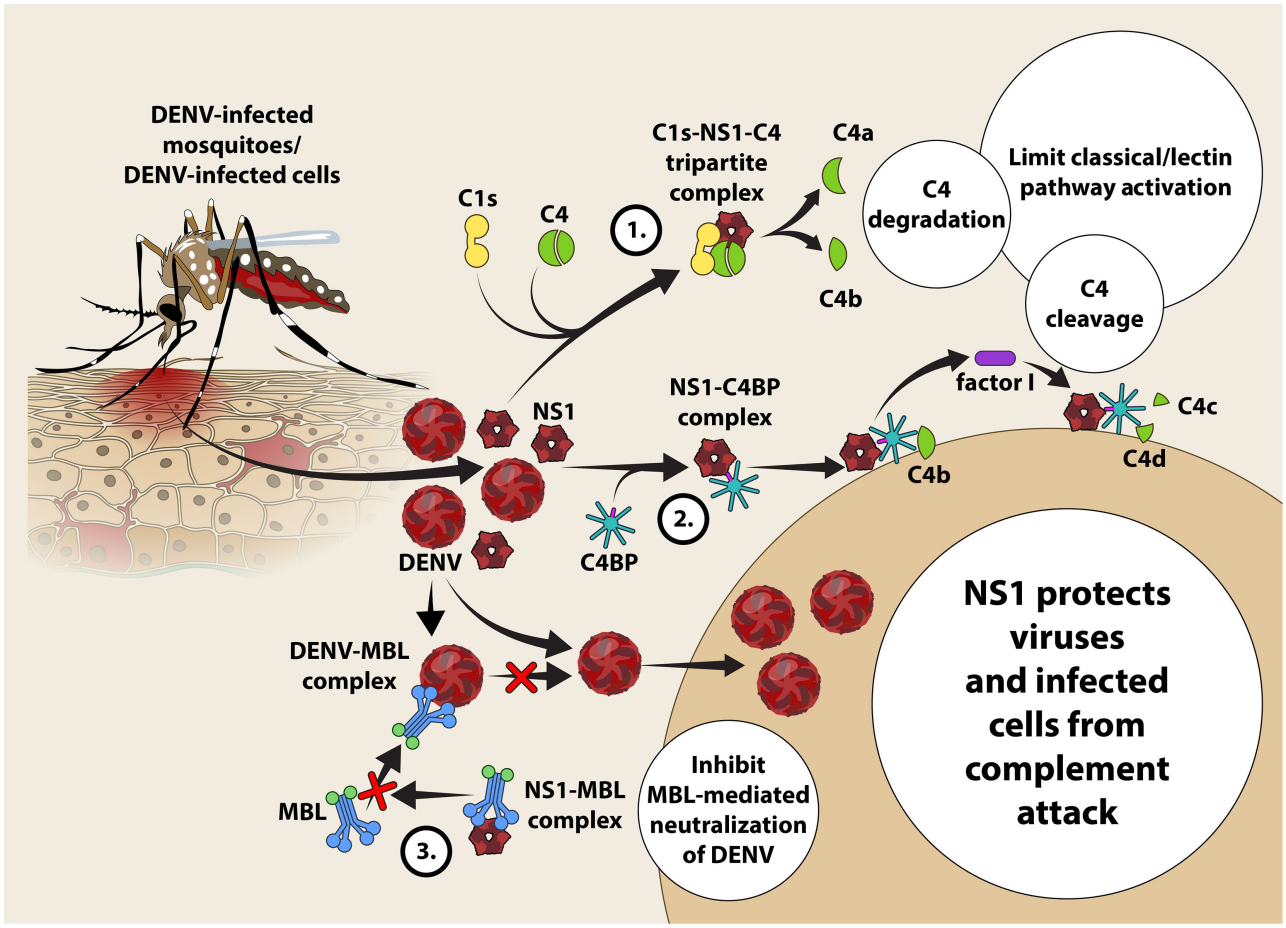
Proposed mechanism of evasion of DENV and DENV-infected cells from classical and lectin pathway-mediated complement attack in the host. NS1 protein released by DENV-infected cells activates the classical pathway of complement activation resulting in the formation of a tri-partite C1S-NS1-C4 complex. C4 undergoes enzymatic cleavage forming C4a (anaphylatoxin and chemotaxin) and C4b present in the fluid phase becomes susceptible to spontaneous hydrolysis attributing to paucity of C4 in the circulation (33). NS1 recruits C4BP and binds with it leading to subsequent recruitment of C4b to engage with the NS1-C4BP complex to allow the ‘stepping-in’ of factor I. Factor I is a negative regulator of complement activation that cleaves C4b into C4c and C4d fragments to limit the classical as well as the lectin pathways (34). Competitive binding of NS1 to MBL prevents recognition of DENV by MBL that protects DENV from neutralization by MBL.

Studies suggest that complement inhibits DENV infection through the classical pathway with C1q binding to E protein and the lectin pathways with MBL binding to virions (47), although DENV has evolved specific counterstrategies to avoid their activation. NS1 forms a multimeric structure that can be found in two forms, either in membrane-bound form (mNS1) or soluble form (sNS1). While mNS1 requires anti-NS1-specific antibodies to activate complement, sNS1 can activate it with or without anti-NS1-specific antibodies. The sNS1 is known to bind C1s and C4, which results in increased cleavage of C4 to C4a and C4b and sNS1 function as a “decoy” to limit the availability of complement to opsonize and lyse the DENV virions (47). Interestingly, sNS1 from DENV-infected insect cell line is able to bind to human MBL, which protect the virions from MBL-mediated neutralization, suggesting the role of NS1 in inhibiting complement activation at the site of the mosquito bite (48). Likewise, sNS1 present in human serum can enhance DENV acquisition by mosquitos (49). sNS1 can also inhibit MAC formation by interacting with vitronectin (VN), clusterin, and the terminal complement protein C9, thereby protecting the infected cells from complement-mediated lysis (47) (**Figure 2**).

## Plasmablasts – Newer Evaluation Yardsticks/Platforms for Clinical Dengue Severity?

Following infection, B cells can be activated by DENV, *via* direct or indirect modes. Despite relatively poor permissibility, B cells are susceptible to DENV infection *via* a phosphatidylserine receptor, CD300a that serves as a portal for virus attachment and entry (46). Coculture of primary B cells with DENV gives rise of substantial amount of IgM release suggesting a heightened level of B cell activation (50). DENV activation of B cells is dependent on CD81 molecule that likely transmits signal *via* the ERK, p38 and JNK MAPK signalling pathways (50). An *in vitro* induction experiment with CD40L, IL-2 and IL-21 revealed that pre-infected B cells display enhanced rate of differentiation into plasmablasts and plasma cells (51). Nonetheless, B cells are not the ideally replicating host cells for DENV as low titers of infectious virions are still detectable in culture supernatants (46). Notably, NS3^+^ B cells could be found in the peripheral blood of acute DENV-infected patients, and other viral antigens can also be detected in the germinal centers of infected individuals (46, 52, 53). This collectively implies that B cells are not only susceptible to DENV infection, but their movement may promote viral dissemination in the body (46). On the other hand, B cells are also indirectly activated through a bystander reaction, by inflammatory mediators released by activated monocytes/macrophages that promote the differentiation of resting B cells into antibody-secreting plasmablasts (54, 55), secreting virus-specific neutralizing antibodies to eliminate the infection.

In the last decade, multiple clinical studies have reported on the expansion of DENV-specific plasmablasts following acute infection (56, 57). Although the phenomenon occurs in both primary and secondary infections, it is transient as the plasmablast population rapidly contracts to baseline level during convalescence (57). Interestingly, the magnitude of plasmablast response is reportedly unique for dengue fever as no such levels of plasmablasts can be seen in the patients with other febrile illnesses or individuals receiving influenza and yellow fever immunizations (56, 58). The total number of CD19^+^IgD^-^CD38^high^CD27^high^ plasmablasts in DENV-infected patients increases strikingly over 1000-fold relative to the basal level in healthy individuals, reaching up to 1×10^6^ plasmablasts per ml of blood within one week after the onset of symptoms (56). During acute DENV infection phase, the percentage of plasmablast population constitutes an average of 30% of the total lymphocyte, and around half of the total peripheral B cell populations (56).

Growing lines of evidence suggest a strong correlation between the number of plasmablasts and dengue severity. In patients with severe dengue, a significantly higher proportion of plasmablasts occupying ~87% of the total peripheral B cell pool has been described (59, 60). The plasmablast response peaks during severe illness and declines soon following recovery, coinciding with disease pathology (56, 61). The highly augmented plasmablast population in severe dengue implies a paramount role in dengue immunopathogenesis. However, further investigations are warranted to unveil role plasmablasts in the setting of DENV infection as differentiated from other viral infections.

### Plasmablast Reactions in DENV Infection

**Figure 3:**
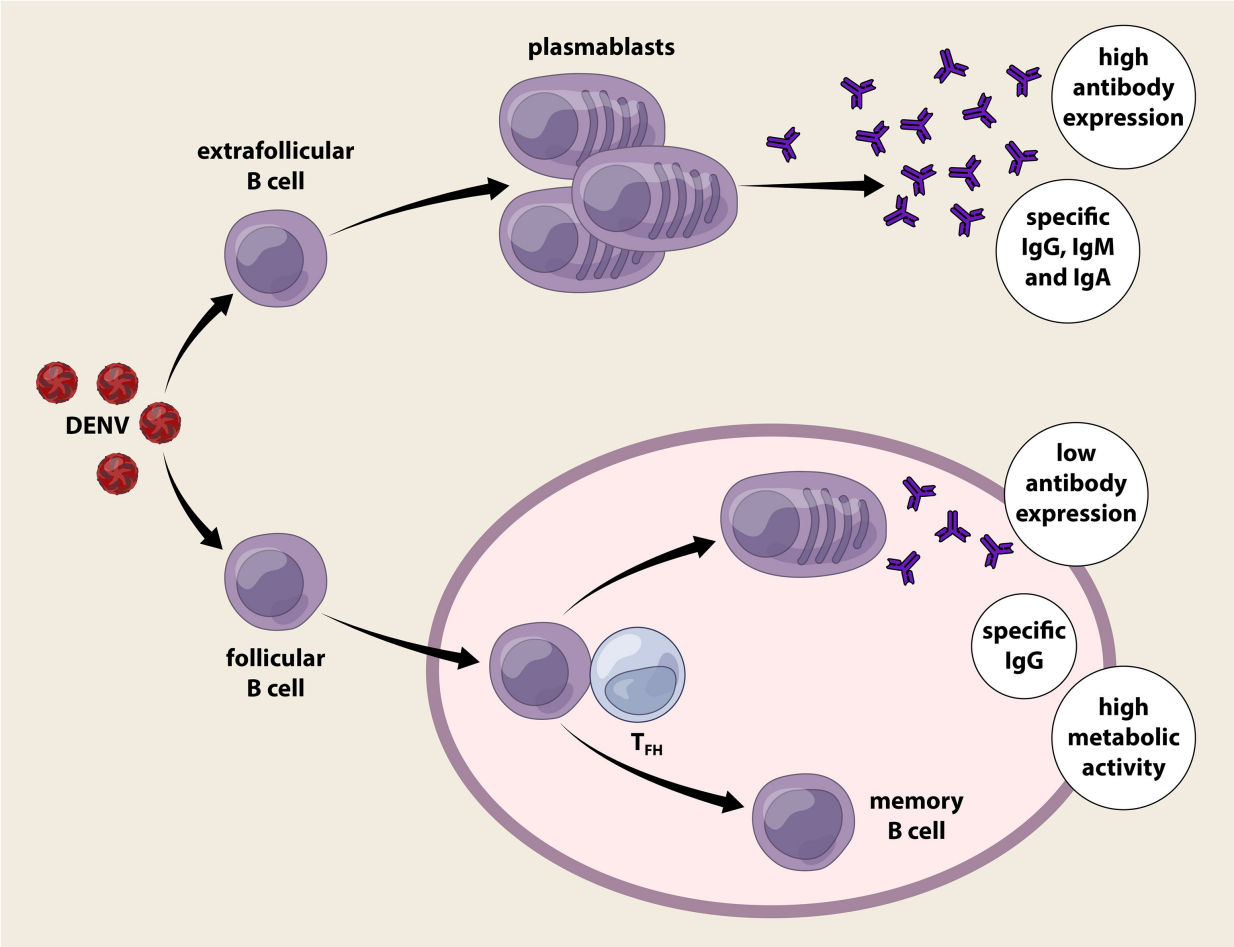
T-dependent and T-independent plasmablast reactions elicited by dengue infection. Dengue virus infection activates B cells directly or indirectly, expanding the B cells into a vast number of circulating plasmablasts. Plasmablasts that are derived from extracellular B cells proliferate rapidly in an innate manner without the requirement of T cell help. The Immunoglobulin gene of this plasmablast population is hypomutated as it lacks germinal centre reaction and produces IgM, IgG and IgA. T-independent plasmablasts response vigorously during acute dengue infection regardless of primary or secondary infection. On the other hand, follicular B cells activated receive help from follicular helper T cells (T_FH_) and initiate the germinal centre reaction where clonal expansion, class switch and active somatic hypermutation (SHM) occurred. The plasmablast produced are long lived and predominant antibodies released are of IgG isotype. Surprisingly, these plasmablasts with evidence of T cell help secrete poor levels of antibodies compared to those without T cell help, but present with high oxidative phosphorylation, eukaryotic initiation factor (EIF2) pathway, and mitochondrial dysfunction. Memory B cells generated following dengue infection provides long term protection after recovery.

B cell activation is driven primarily by antigen recognition through its surface B cell receptor (BCR) following infection or vaccination, which occurs either extrafollicular in a T-cell independent, or follicular in a T-cell dependent manner. Short-lived plasmablasts arise from extrafollicularly-activated B cells, whereas follicular B cells diverge into two distinct lineages, i.e., antibody secreting plasmablasts or memory B cells following T-dependent cell activation. Circulating plasmablasts constitute the major source of antibodies critical for virus clearance, whereas memory cells provide protection from secondary infection by differentiating robustly into antibody-secreting plasma cells. Plasmablast response in the context of DENV is characterized by concurrent T-dependent and T-independent B cell responses (62, 63) (**Figure 3**). High-throughput cDNA sequencing of the human Ig gene heavy chain variable region (V_H_) suggests a low percentage of plasmablasts displaying somatic hypermutated antibody genes even during secondary infection or severe dengue (62). The dominant population with hypomutated V_H_ segments likely represents extrafollicular B cell-derived plasmablasts independent of T cells. Despite the lack of T cell help, the T-independent plasmablasts actively secreting antibodies particularly of IgG isotype with high neutralizing ability has been reported even after primary infection (57, 61). This suggests that germ-line encoded Ig genes give rise to poly-reactive antibodies that functions during acute dengue infection (62). By contrast, a high degree of somatic hypermutation in dengue plasmablasts has been reported, highlighting the role of T-dependent activity (64). A sub-optimal T cell response compared to B cell response occurs normally in dengue infection (65). Hospitalization is reportedly associated with lower frequencies of activated, terminally differentiated T cells and higher percentages of effector memory CD4^+^ T cells (61). Follicular helper T cells (Tfh) increase during acute DENV infection, peak at critical disease phase, and are higher during secondary compared to primary dengue (66, 67). Tfh cells express follicular homing chemokine receptor CXCR5, PD-1, ICOS and produce IL-21, which support the B cells in germinal centre (68).

The percentage of Tfh also positively correlates with the frequency of plasmablasts and the titre of specific antibodies against DENV (66, 68). Surprisingly, the plasmablasts receiving T cell help following naïve B cell activation secrete low antibody levels, but have high oxidative phosphorylation, activation of eukaryotic initiation factor (EIF2) pathway, and mitochondrial dysfunction (63). A recent longitudinal analysis of DENV-infected patients has revealed distinct antibody responses and B cell activation during primary and secondary responses, suggesting the role of memory B cells in secondary dengue (61). Serotype-specific antibodies are retained in patients six-months post-infection and the serum antibody concentration correlates with the percentage of specific memory B cells (61, 69). Others have reported that **~**8% of DENV-specific CD27^+^CD38^-^ memory B cells undergo class-switching during acute and early convalescence (70). In fact, memory B cells secrete antibodies for a longer duration even after two decades post-infection (71). Despite this, plasmablasts and memory B cells in DENV infection are poorly linked to each other (72). Plasmablast expansion during acute dengue does not seem to be directly derived from memory B cell clones or contribute to memory B cell repertoire during convalescence. The preference of Ig heavy chain usage and complementarity-determining region (CDR3) length differs between plasmablasts and memory B cell clones (72). DENV-specific memory B cell clones predominantly express IgM, and likely requires a fresh round of germinal centre reaction during reinfection; whereas the plasmablasts that appear rapidly following infection predominantly express IgG (72).

## Interplay Between DENV, Inflammasome, Complement Activation, Auto-Reactive Antibodies and Cytokine Storm in Endothelial Dysfunction

### Vascular Leakage Induced by NS1

The hallmark of severe dengue is the transient endothelial dysfunction leading to increased vascular permeability as well as the alteration in the coagulation cascade leading to bleeding, pleural effusion and hypovolemic shock (73). Although dengue pathogenesis is not completely understood, DENV NS1 is regarded as the “viral toxin” and the key mediator for severe disease. Firstly, NS1 activates the complement cascade leading to exaggerated release of vasoactive anaphylatoxins, which in turn cause aberrant activation of mast cells and histamine release leading to increased vascular permeability and endothelial dysfunction. This is evidenced by the presence of high levels of NS1, C3a, C5a and in the plasma of DHF patients before plasma leakage and in the pleural fluids of DSS patients, and their levels correlating with disease severity (47, 74). Secondly, the anti-NS1 antibodies (IgM and IgG) form complexes with both mNS1 and sNS1 leading to complement-dependent lysis of the host cell and ADCC, damaging the endothelial layer to necessitate vascular leakage (47). Furthermore, sNS1 also engages with TLR4 expressed on monocytes, macrophages and endothelial cells, leading to cytokine storm, further damaging the endothelium (75).

### NS1-Induced Coagulopathy

Bleeding tendency, plasma leakage and thrombocytopenia are the key clinical features of DHF/DSS. DENV infection induces polyclonal and highly cross-reactive antibodies (46, 50, 64). Studies have demonstrated that anti-NS1 antibodies are autoreactive, targeting various epitopes present on human including platelets and other proteins related to coagulation process (e.g. fibrinogen, plasminogen, thrombin) as well as extracellular matrix proteins of endothelial cells (76). During dengue infection, blood vessels may be injured due to autoreactive ADCC against mNS1. Activated platelets adhere to the injury site followed by changing shape, releasing granule contents, and eventually aggregating together through fibrin formation. It has been reported that the concentration of NS1 proportionally correlated with the disease severity (12, 15, 77) and could be as high as 50 μg/ml among DHF/DSS patients. During the defervescence, these NS1 is cleared from the circulation by antibody-mediated respond. Countless autoreactive antibodies are attacking self-component during the course of clearing NS1 (78–81) and the coagulation process is inhibited; further leading to bleeding, plasma leakage and shock. This is partly explain why plasma leakage is often happen during the defervescence phase. In a recent meta-analysis, the study showed that the activated partial thromboplastin time (APTT) and prothrombin time (PT) are significantly prolonged among patients with dengue infection where the APTT and PT were 42.91% (95% CI: 30.95, 54.87) and 16.48% (95% CI: 10.95, 22.01), respectively (82) compared to uninfected individuals, rendering them more prone to develop bleeding manifestations during dengue disease. NS1 has also been reported to form immune complexes with erythrocytes leading to complement-induced erythrocyte damage (76, 83).

### Vascular Leakage Induced by Inflammasome – Role of NS2a and NS2b

**Figure 4:**
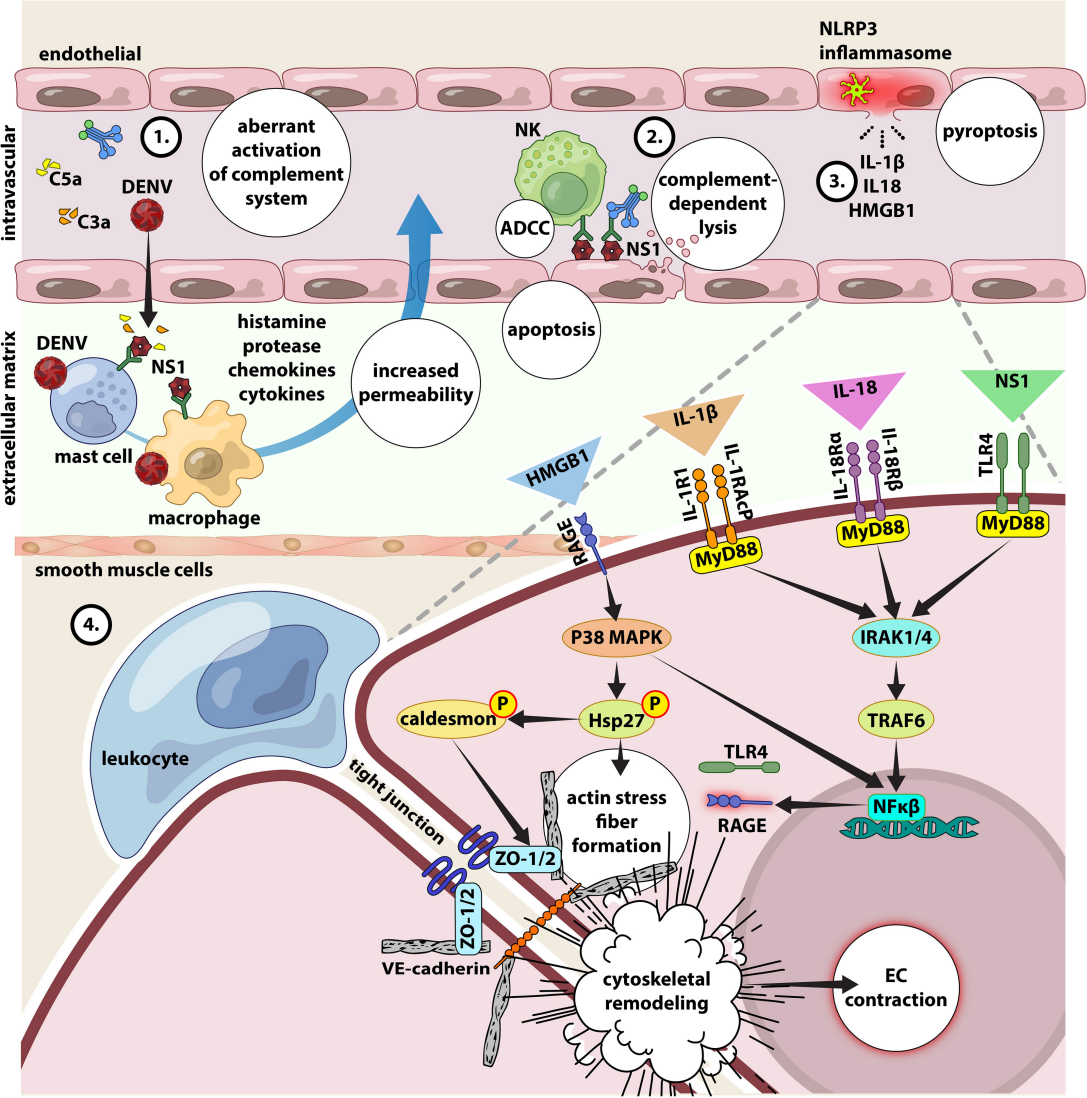
Interplay between NS1, inflammasome activation, complement activation, autoreactive antibodies and cytokine storm in endothelial dysfunction. 1) DENV and NS1 can directly activate CS, the excessive conversion of C3, C4 and C5 to their active forms C3b, C4b and C5b will inevitably cause increased level of C3a, C4a and C5a (anaphylatoxin). Large amount of anaphylatoxin will cause aberrant activation of mast cells and release massive amount of histamine, along with other pro-inflammatory cytokines, leading to increase of the vascular permeability and vascular leakage. 2) anti-NS1-specific IgM and IgG have been found circulating in the blood stream forming immune complexes with the both forms of NS1 proteins. While mNS1 is expressed on DENV infected cells, the circulating sNS1 can subsequently bind to surface of infected or uninfected cells *via* heparan sulfate and chondroitin sulfate E (90). These antibodies will then bind to NS1 and leading complement-dependent lysis of cell and antibody-dependent cellular cytotoxicity, damaging the endothelial layer and leading to vascular leakage (47). 3) Secretion of IL-1β, IL-18 and HMGB1 results from NLRP3 inflammasome activation. 4) In the circulation, IL-1β and IL-18 binds to their cognate receptors (IL-1R1/acP and IL-18Rα/β) expressed on the surface of endothelial cells to activate intracellular signaling molecules, involving MyD88, IL-1 receptor-associated kinase 1/4 (IRAK1/4), and TNF receptor-associated factor (TRAF), which entails in NF-κB activation. NS1 can also activate the NF-κB signaling pathway *via* TLR4. The activation of NF-κB signaling pathway increases the secretion of pro-inflammatory cytokines and chemokines to mediate leukocyte adhesion and extravasation (diapedesis). Additionally, the binding of HMGB1 to the RAGE receptor leads to the downstream activation of p38 MAP kinase, resulting in phosphorylation of the actin-binding protein Hsp27 and caldesmon, which causes actin stress fibers to form, cytoskeletal remodeling, and endothelial contraction. All these reactions increase endothelial permeability by altering cell contractility and disruption of intercellular junctions. Given the ability of IL-18 to induce the production of various pro-inflammatory mediators including TLR4 and RAGE *via* activation of NFκ-B signaling (75, 91, 92), their higher expression will condition the endothelial cells to be more responsive to stimulation by NS1 and HMGB1, to further magnify inflammation and vascular permeability.

Monocytes are susceptible to DENV infection and produce several key proinflammatory cytokines contributing to endothelial dysfunction and vascular leakage. IL-1β, IL-18, IL-33 and high-mobility group B1 (HMGB1) represent the key inflammatory mediators produced during hypercytokinemia, *via* the inflammasome pathway and have been shown to correlate with dengue severity (20, 84, 85). Given that NS2A and NS2B can trigger the activation of NLR family pyrin domain containing 3 (NLRP3) (86), the main component of an NLRP3 inflammasome, the more the cells infected by DENV *via* ADE, the higher the release of IL-1β, IL-18 and HMGB1 (87). IL-1β and IL-18 are produced *via* the NLRP3 inflammasome-caspase-1 pathway. They bind to their cognate receptors (IL-1R1 and IL-18Rα/β) to promote Th1 cell activation, cytotoxic functions of CD8+ T cells and NK cells, release of cytokines, especially IFN-γ along with other mediators of inflammation namely IL-2, IL-12, TNF, and GM-CSF. IL-18 also induces the expression of chemokine and adhesion molecules on endothelial and smooth muscle cells (88). HMGB1 is, when released from cells, an ‘alarmin’ in peripheral circulation during pyroptosis, a pro-inflammatory cell death pathway induced by aberrant activation of inflammasome, and hence is a byproduct of inflammasome activation. In the circulation, HMGB1 binds to its cognate receptor, receptor of advanced glycation end-products (RAGE) resulting in the activation of an intracellular pathway that culminates in the formation of actin stress fibers, remodeling of cytoskeleton, contraction of endothelium and vascular leakage (89). Because RAGE is abundantly expressed along the lung endothelium, this likely could be the attribute behind why plasma leakage often manifests as pleural effusion in the lungs (89) (**Figure 4**).

## DENV and SARS-CoV-2 Coinfection – Pathogenic Synergism or Antagonism?

Since December 2019, coronavirus disease 2019 (COVID-19) caused by severe acute respiratory syndrome coronavirus 2 (SARS-CoV-2) has wreaked havoc globally and the pandemic continues to be a public health concern. Because certain features of clinical and laboratory presentation of dengue and COVID-19 seems to be overlapping, the correct diagnosis and treatment of co-infection is challenging, especially in tropical regions where they co-exist (93, 94). **Table 1** shows the similarities between DENV and SARS-CoV-2. It is to be noted that both DENV and SARS-CoV-2 have similar incubation periods presenting with acute febrile phases. In such cases, respiratory symptoms such as cough and dyspnea should raise clinical suspicion towards COVID-19. While significant thrombocytopenia is a prominent feature observed in dengue, it is less common for COVID-19, reported in only about 5 to 12% of cases (94). A case report describes a patient with concurrent positivity for dengue serology and COVID-19 (by RT-PCR) raising concern on the possibility of co-infection with both viruses considering the dengue-endemic geography region of reports and call for rapid and sensitive diagnostic tests (105, 107). In a systematic review conducted between January and November 2020, it has been concluded that COVID-19 and dengue co-infection was associated with severe disease and fatal outcomes (108).

**Table 1:** Similarities in characteristics between dengue infection and COVID-19.

S. No.	Characteristic features	References
1.	Both DENV and SARS-CoV-2 possessing ssRNA as genetic material	(95, 96)
2.	Similar incubation period with acute febrile illnesses during the early stages of infection	(97)
3.	Pathophysiological presentations like capillary leakage and coagulopathy	(98)
4.	Presentation of skin manifestations and petechiae	(89, 93)
5.	Elevated levels of CRP, D-dimer and IL-6	(99–102)
6.	Antibody-dependent enhancement	(103, 104)
7.	Possible antigenic cross-reactivity between DENV and SARS-CoV-2	(105, 106)

Organizing pneumonia, the prominent complication in COVID-19 patients results from lung injury caused by a surge in levels of proinflammatory cytokines. Various studies have demonstrated the correlation of hypercytokinemia with the breadth of lung injury, multiorgan failure, ICU admission and poor survival rates (especially with IL-6 levels) in COVID-19 patients (95–97). Cytokines such as IL-6, IL-2, IL-7 and many others have been demonstrated to be associated with the severity of COVID-19 (98). Similarly, elevation in the levels of proinflammatory cytokines leading to hypercytokinemia is observed in patients with DHF attributing to vascular leakage and disease pathogenesis (99–101). The role of hypercytokinemia in the dengue pathogenesis, similarities in certain clinical presentations and pathological changes in multiple organs are shared between DENV and SARS-CoV-2 (102). In both DENV and SARS-CoV-2 infections, elevated levels of proinflammatory cytokines and chemokines such as IL-6, IL-1β, IL-8, CXCL9, MCP-1 and IP-10 as well as IL-10 have been positively correlated with the severity of clinical illness (99, 103, 104, 106). A study that compared the responses in patients with varying severity of COVID-19 and acute dengue infection at different time-points showed significantly higher levels of IL-6, IL-10 and MIP-3α among those who developed COVID-19 pneumonia and DHF than those with mild manifestations (102). Interestingly, the finding points at the elevated levels of IL-10 in both COVID-19 as well as DENV infection during the early phases of illness as indicators of altered immune responses possibly lead to severe disease presentation and poor prognosis.

A recent study used a series of computational analyses to build host factor interaction networks and elucidated the biological process and molecular function categories for a better understanding of the underlying mechanisms of SARS-CoV-2 and DENV coinfection. Of the 460 shared host factors identified, CCL4 and AhR targets were significantly upregulated, and the study also demonstrated the role of the NOD-like receptor (NLR) and TLR signaling pathways during the interaction of the viruses with the host. The study reported that *IL-1β, Hippo, p53, TNF-α* and *TLR* were significantly upregulated. Furthermore, the tissue-specific enrichment analysis revealed an increase in the expression of ICAM-1 and CCL2 in the lungs (109). It has also been reported that SARS-CoV-2 and DENV coinfection could result in severe pulmonary impairment, hospitalization rates and grave clinical outcomes (110).

It also appears that the serological cross-reactivity between DENV and SARS-CoV-2 can have serious implications in the diagnosis point-of-view and can pose a diagnostic predicament in geographical locations endemic to dengue. A study from Singapore had reported a couple of cases with false-positive results for dengue based on rapid serological assays, which were later confirmed as COVID-19 positive (111). In a report from Thailand (where dengue infections are common), a COVID-19 patient presenting with petechial rash and low platelet count was initially misdiagnosed as dengue (112). A study from Indonesia had also reported false-positive results between dengue and COVID-19 despite the high specificity of the rapid diagnostic tests for COVID-19 (113). An interesting study from India wherein rapid dengue antibody detection tests were performed on archived serum samples pre-dating the COVID-19 pandemic and those positive for dengue were subjected to SARS-CoV-2 detection. Of the thirteen dengue positive sera tested, five produced false-positive results in SARS-CoV-2 lateral flow-based rapid tests. These results demonstrate the possibility of cross-reactivity and false-positive COVID-19 results in dengue-endemic countries like India. Interestingly, the study has also demonstrated that antibodies against DENV can cross-react with the receptor-binding domain (RBD) of SARS-CoV-2 spike protein based on computational analysis (114). A recent investigation from Brazil investigated 2351 COVID-19 patients to report that those with a history of dengue infection had a lower risk of mortality hinting the likely protection induced by DENV against severe COVID-19, which nevertheless remains a component warranting rather more evidence (115). It appears that although coinfection with SARS-CoV-2 and DENV combination can aggravate the clinical severity alongside diagnostic predicaments (in dengue-endemic areas), the interaction between the viruses remains a grey area of investigation. Further, the immune correlates of protection in DENV-SARS-CoV-2 coinfection needs thorough investigation, and must explore the possibilities of identification of improved therapeutic targets.

## A Call to Newer Interventions Against Dengue Disease

While our efforts to develop a safe and effective dengue vaccine are progressive albeit for many decades, we are yet to achieve significant success at these quarters. The live-attenuated, tetravalent DENV candidate vaccine developed by Sanofi Pasteur Inc., Swiftwater, PA, USA has shown varying levels of protective efficacy against all the four different DENV serotypes, viz., DENV1 (61.2%), DENV3 (81.3%), and DENV4 (89.9%), whereas no protection against DENV2 (116) has been reported. Owing to ADE (117), an increased risk of hospitalization and development of severe dengue among DengVaxia^®^ recipients has been observed during the four Phase 3 clinical trials (viz., CYD17, CYD29, CYD32, and CYD33) conducted in the Philippines in 2019 (118). Perhaps vaccines are not the “*silver bullets*” against development of dengue disease (also with COVID-19). With the extensive understanding we have had on the immunopathogenesis of dengue disease, perhaps there are other potential interventions focusing on harnessing passive immunity, host-directed immunomodulation as well as alleviation of platelet loss, which likely could add to amelioration of dengue disease severity.

### Passive Immunity Approaches Against Dengue

Recently, the U.S. Food and Drug Administration revised the emergency use authorization (EUA) for two neutralizing monoclonal antibodies, Bamlanivimab and Etesevimab for the emergency therapy against mild to moderate COVID-19 in pediatric patients, including neonates, who had a positive COVID-19 test and were also at an increased risk for development of severe COVID-19 (119). Similarly, monoclonal antibodies directed against all the four DENV serotypes could be developed and administrated in accordance with the infecting serotype in the corresponding group of patients. Such interventions where the corresponding drug is administered at optimum neutralizing levels, may effectively mitigate the risk of development of ADE in dengue patients.

Secondly, since DENV NS1 has been regarded as viral toxin implicated in the aberrant activation of the complement cascade, excessive release of anaphylatoxins C3a and C5a leading to eventual onset of increased vascular permeability and endothelial dysfunction, the neutralization of NS1 appears to be a promising strategy to reduce the severity of dengue illness and complications. Several lines of evidence show that the anti-NS1 antibodies can effectively block the NS1 from interacting with the surface of endothelial cells, thereby limiting the NS1-induced disruption of the intercellular junction complex (31, 120, 121).

More recently, by using a mouse model O’Donnell et al. (2020) demonstrated that the polyclonal avian IgY generated against a suspension of whole, killed DENV2 can confer protection against a lethal challenge of dengue infection without the development of ADE. This appears to be attributed to IgY’s ability to neutralize DENV notwithstanding the avian Fc does not bind with the mammalian Fc receptor (122). The above experiment provides us a viable clue for a promising therapeutic approach against DENV. Nonetheless, since anti-NS1 antibodies are known to be autoreactive and linked to coagulopathy and endothelial dysfunction (76), the use of anti-NS1 antobodies in treating dengue disease must be carefully selected to avoid antoreactivity. Further, with significant advances in antibody engineering, the Fc domain can be fine-tuned in such a way that it does not induce ADE or autoreactivity (123, 124).

### Immune Modulation in DENV Disease

#### A) Anti-C5 mAb

It is now clear that exaggerated activation of the complement cascade could contribute to dengue severity and vascular leakage, and hence drug interventions that can regulate complement activation would likely be a valid target candidate. Eculizumab is an anti-C5 mAb that was initially approved to treat **atypical hemolytic uremic syndrome** (aHUS) (125, 126). Because the vasculopathy in dengue is mainly due to excessive release of C3a and C5a, the potential use of anti-C5 mAb may likely reduce vascular leakage and associated complications in severe dengue (127).

#### B) MCC950

Having said that aberrant activation of inflammasome, especially NLRP3 inflammasomes could play an important role driving the onset of cytokine storm, inhibitors/blockers of the inflammasome would be an alternative promising approach against severe dengue-associated manifestations. MCC950 is a highly potent small molecule inhibitor of NLRP3, and binds directly to the NATCH (NAIP, CIITA, HET-E, and TP-1) domain of NLRP3 preventing it from undergoing conformational changes and assembly (128, 129). *In vivo* studies have shown that MCC950 reduced IL-1β production and attenuated the severity of experimental autoimmune encephalomyelitis (EAE) (130). Nonetheless, the applicability of treating clinical dengue with MCC950 has seldom been examined warranting in-depth investigations in the future.

#### C) Targeting IL-18

IL-18 is an important (Th1 polarizing) cytokine produced during pyroptosis by the activation of NLRP3 inflammasome alongside IL-1β. However, the biological activity of IL-18 is regulated by a regulatory protein, named IL-18 binding protein (IL-18BP). IL-18BP is a natural-occurring soluble molecule that binds to IL-18 with a higher affinity, preventing IL-18 from engaging with the IL-18R expressed on cell surfaces, thereby regulating the bioavailability of IL-18 to other lymphocytes (131). In a previous study, we compared the plasma level of IL-18 and IL-18BP among dengue patients with and without warning signs as well as severe dengue. We also determined the bioavailability of IL-18, and found that the plasma levels of IL-18 was significantly higher in patients with severe dengue followed by those with dengue with warning signs as well as dengue without warning signs. However, the levels of IL-18BP were not significantly different. Having said that, the bioavailability of IL-18 is higher among patients with severe dengue as well as among those with warning signs as compared to dengue without warning signs (84). This suggests that the cytokine storm occurring in severe dengue disease may be due to inadequate regulation of IL-18 responses by IL-18BP.

In a more recent study, Li et al. (2020) used recombinant human IL-18 (rhIL-18BP) to decrease soluble IL-18 in irradiated experimental mouse. The administration of IL-18BP was found to inhibit the down-stream activities of IL-18, and the treated mouse showed low levels of IFN-γ, reactive oxygen species (ROS) as well as attenuated a stress responsive factor named growth differentiation factor-15 (GDF-15) (132). The study was encouraging as it showed that IL-18 played a paramount role in radiation-induced tissue damage and treatment with IL-18BP likely mitigates tissue injury/damage induced by radiation. Hence, the use IL-18BP appears to serve as a promising therapeutic strategy against severe dengue disease warranting investigations.

### Reducing Platelet Loss

Thrombocytopenia and platelet dysfunction are commonly observed in DENV patients and dengue complications are usually preceded by a rapid drop in platelet count (133). Platelet, traditionally known for their key role in coagulation, but nowadays are well known to have important additional functions, including regulation of inflammation and host defense (134) and preservation of endothelial integrity (135), especially under inflammatory conditions (136). Circulating platelets of dengue patients are found to be over activated and exhausted (137), which likely contributes to platelet loss and coagulopathy. Other well-known mechanisms of platelet loss is the binding of von Willebrand factor (VWF) to platelets and loss of sialic acids from the platelet membrane (138). VWF is a multimeric protein that is present in plasma, platelets, and endothelial cells. Endothelial activation results in the release of VWF from Weibel-Palade bodies into the blood circulation with its primary function is to allow platelet adhesion and aggregation at sites of vessel injury (139). As dengue infection can cause endothelial activation, massive amount of VWF are released into circulation. Study showed that the VWF level was elevated in dengue infection and particularly heighten among DSS patients (139). Mechanistic studies revealed that the binding of VWF to platelets results in translocation of neuraminidase to the platelet membrane and subsequent cleavage the sialic acid off from the membrane (140). The good news is that this process could be inhibited by the neuraminidase inhibitor oseltamivir, or more commonly known as Tamiflu, the anti-influenza drug (141). The efficacy of oseltamivir in preventing thrombocytopenia in dengue disease should be investigated. The newly proposed approaches aforementioned above are summarized in **Table 2**.

**Table 2:** Interventions for dengue disease and their stage of development.

Vaccines and other DENV specific therapies			
Product name		Stage	Outcomes	
**CYD-TDV** – recombinant tetravalent dengue vaccine developed by Sanofi Pasteur given as a 3-dose series on a 0/6/12 months schedule.		Phase III clinical trials. (not prequalified)	Varying efficacy against different serotype of DENV where DENV1 (61.2%), DENV3 (81.3%), and DENV4, (89.9%) whereas no protection against DENV2 (116). There is an increased risk of hospitalized and severe dengue in seronegative individuals starting about 30 months after the first dose (142).	
Serotype-specific anti-DENV monoclonal Abs		N.A.	N.A.	
Anti-NS1 mAb		Animal study	Administration of NS1-mAb to mice as late as 1 day prior to severe bleeding occur still reduced hemorrhage (121).	
Anti-DENV avian **IgY**		Animal study	Confer protection against a lethal challenge of dengue infection without the development of ADE. The Abs also cross protective to ZIKA virus (122).	
**Repurposing interventions for treating DENV infection**			
**Product name**	**Initial purpose**	**Stage**	**Drug action & rationale or repurposing**
Eculizumab	To treat atypical hemolytic uremic syndrome (aHUS)	FDA approved	Anti-C5 Abs (inhibitor to C5) C5 is anaphylatoxin Excessive production of C5 can lead to vascular leakage.
MCC950	Regulate inflammation related to NLRP3	Animal study	Reduced IL-1β production and attenuated the severity of experimental autoimmune encephalomyelitis
		Phase II	Rheumatoid arthritis Clinical trial failed due to high liver toxicity (143).
rhIL-18BP	Regulate inflammation related to over production of IL-18	Animal study	IL-18BP counters IL-18 activity after expose to lethal dose of radiation exposure. Able to mitigate radiation-induced multiple organ injuries and increase animal survival (132).
Oseltamivir (Tamiflu)	Anti-influenza medication	FDA approved	Neuraminidase inhibitor Reduce the activity of neuraminidase in cleaving sialic acid away form membrane of platelet, thereby protect platelet from over activation.

NA, not available.

## Concluding Remarks and Future Perspectives

The dengue pandemic was initially reported in Southeast Asia after World War II and has spread rapidly to almost all tropical countries. Dengue poses an enormous burden to public health systems worldwide as >40% of the global population is at risk of infection. Following infection, viruses undergo replication in the local tissues such as the skin, which leads to an activation of a cascade of events including the recruitment of skin resident cells, e.g., Langerhans cells, mast cells, and keratinocytes, and new cells, e.g., T cells and neutrophils, into the site of the infection. After infection of target cells, sensing of viral products results in the activation of innate immune responses, which establish the inflammatory and antiviral state intended to prevent the virus to replicate and spread. However, DENV utilizes several mechanisms to hijack these responses and escape from the normal immune recognition and processing, which results in its dissemination into the lymph nodes. There, DENV further replicates in monocytic cells, resulting in a primary viremia after its systemic disseminated through the circulatory bloodstream. This results in the subsequent infection of peripheral tissues, such as the liver, spleen, and kidney. Evidence also strongly supports the involvement of multiple cell death pathways following DENV infection leading to vascular dysfunction brought about by monocyte activation. Improved understanding of cell death pathways induced by DENV will help in the development of novel modalities of prevention of disease progression.

In endemic areas where multiple DENV serotypes circulate, distinct epidemiological studies found that an individual can become exposed to and can have sequential infections with distinct DENV serotypes, which poses a risk of developing severe manifestations such as DHF/DSS. This phenomenon has been attributed to the potential enhancement activity that the pre-existing antibody response elicited from a previous infection with one serotype may have on the infection with a different serotype. This process leads to an increased viral burden that triggers a series of immunological and cellular events, e.g., ADE, hypercytokinemia, skewed T cell responses, and complement pathways, which despite being intended to prevent the viral invasion and infection, can induce host tissue damage leading to pathology and disease. Hence, it is important to study the immunopathology of dengue fever, as we gain more insights into the pathogenic mechanisms of DENV infections, we can hope to improve our efforts towards providing better case management, reduce its overall morbidity and mortality, and assist in the development of safe and effective vaccines against the dreadful disease.

## Clinician’s Corner

Maintenance of adequate hydration is key to dengue management. Patients also must be monitored for warning signs of severe dengue disease, and hence, prompt initiation of early management/treatment intervention is key to preventing dengue-associated complications such as prolonged shock and metabolic acidosis. Hence, the mainstay of successful management includes judicious and timely initiation of IV fluid replacement therapy with isotonic solutions and frequent monitoring of the hemodynamic status and vital signs during the critical phase. Patients should be administered with acetaminophen for pain as well as temperature management. Aspirin and non-steroidal, anti-inflammatory medications could aggravate the bleeding tendency in some patients and, in children, can be associated with the development of Reyes syndrome.

## Author Contributions

YKY, WFW, RV, IC, and EMS wrote the manuscript. YKY and EMS critically revised the article for important intellectual content, and approved publication of the article. VV, YZ, ML, and HYT provided critical inputs to the manuscript. All authors contributed to the article and approved the submitted version.

## Funding

This work was supported by Xiamen University Malaysia Research Funding (XMUMRF), (XMUMRF/2018-C2/ILAB/0001) to YKY, (XMUMRF/2020-C5/ITCM/0003) to HYT and (XMURF/2018-C1/ENG/0005) to YZ. VV was supported in part by NIH R01AI148377 (to VV), Emory University CFAR grant P30 AI050409, NIH Office of Research Infrastructure Programs (ORIP) grants P51 OD011132 and U42 OD011023 (to ENPRC). The Swedish Research Council, The Swedish, Physicians against AIDS Research Foundation, The Swedish International Development Cooperation Agency; SIDA SARC, VINNMER for Vinnova, Linköping University Hospital Research Fund, CALF, and The Swedish Society of Medicine (AI52731) to ML, Funding support provided by the Department of Science and Technology-Science and Engineering Research Board, Government of India (CRG/2019/006096) (to EMS).

## Author Disclaimer

The content is solely the responsibility of the authors and does not necessarily represent the views of the official affiliations of the authors.

## Conflict of Interest

The authors declare that the research was conducted in the absence of any commercial or financial relationships that could be construed as a potential conflict of interest.

## Publisher’s Note

All claims expressed in this article are solely those of the authors and do not necessarily represent those of their affiliated organizations, or those of the publisher, the editors and the reviewers. Any product that may be evaluated in this article, or claim that may be made by its manufacturer, is not guaranteed or endorsed by the publisher.

## Glossary

**Alarmins:** Also called as danger-associated molecular patterns, such as IL-33, Hsp70, HMGB1 and IL-1α, are released by injured or necrotic cells

**Antibody-dependent cellular cytotoxicity:** ADCC is an immune mechanism where an FcR-bearing effector cell recognizes and binds to Fc fragment of an antibody molecule already bound to an antigen displayed on a target cell (for instance, a host cell expressing pathogen-derived antigens on its surface), which entails in downstream signaling within the effector cell resulting in cytolytic granule release and consequent lysis of the target cell *via* the perforin-granzyme pathway.

**Antibody-dependent enhancement:** ADE represents a mechanism where a non-neutralizing antibody becomes bound to a viral particle (often during secondary infection) is recognized by the Fc gamma receptor IIa (FcγRIIa) expressed on a phagocytic cell leading to enhanced intracellular viral replication, or immune complex formation resulting in hyperinflammation and immunopathology. ADE occur when binding antibodies (non-neutralizing) or antibodies at sub-optimal levels bind to viral particles without necessitating viral clearance.

**Atypical hemolytic uremic syndrome:** A rare life-threatening disease resulting from dysregulated activation of the complement system culminating in the formation of thrombi in small blood vessels of visceral organs especially the kidneys progressing to end-stage renal disease (ESRD).

**Cytokine:** A wide range of proteins secreted by various host cells especially immune cells that facilitate intercellular signaling, and can exert localized or systemic biological effects.

**Cytokine storm:** A state of excessive systemic inflammation involving dramatically elevated levels of proinflammatory cytokines and inflammatory cells viz. macrophages, neutrophils, mast cells, eosinophils and basophils. Cytokine release syndrome can often result in the dysfunction of secondary organs, systemic multi-organ failure, and can be fatal.

**Damage-associated molecular patterns (DAMPs):** DAMPs are molecules released by injured and necrotized cells usually following an infection in the host, and are recognized by pattern recognition receptors (PRRs) expressed on host cells to activate innate immune responses.

**Defervescence:** A phase in dengue fever where the patient’s body temperature decreases rapidly.

**Dengue hemorrhagic fever:** DHF is currently defined by four WHO criteria viz., (1) Fever or recent history of fever lasting 2–7 days (2) Any hemorrhagic manifestation (3) Thrombocytopenia (platelet count of <100,000/mm3 (4) Evidence of increased vascular permeability. Common manifestations include a positive tourniquet test, skin hemorrhages (petechiae, hematomas), epistaxis, gingival bleeding, and occult hematuria. (https://www.cdc.gov/dengue/resources/denguedhf-information-for-health-care-practitioners_2009.pdf).

**Dengue shock syndrome:** DSS is defined as any case that meets the four criteria for DHF together with evidence of circulatory failure manifested by (1) rapid, weak pulse and narrow pulse pressure (≤20 mmHg [2.7 kPa]) or (2) hypotension for age, restlessness, and cold, clammy skin. Dengue can rapidly progress into DSS, which, if not diagnosed and treated promptly, can result in far-reaching consequences.

**Experimental autoimmune encephalomyelitis:** EAE refers to an autoimmune form of multiple sclerosis induced in animals resulting from aberrant activation of NLRP3 inflammasome as well as T cells against CNS myelin.

**Fc receptor:** Receptors expressed on certain cells of the host immune system, such as phagocytes, granulocytes, and lymphocytes (NK cells and B cells). These receptors permit the binding of the cells to the Fc regions of antibodies that have become bound to pathogenic surfaces or infected cells, rendering their timely elimination.

**Hemodynamic shock:** HS is an acute syndrome characterised by inadequate blood flow throughout the body leading to organ failure due to poor perfusion. Shock results from a fall in cardiac output (CO) (for e.g. cardiogenic shock, mechanical shock, hypovolaemic shock) or an ebb in total peripheral resistance (TPR) (distributive shock such as toxic/septic shock and anaphylactic shock. A fall in CO or a poor TPR results in low blood pressure culminating in decreased tissue perfusion and multi-organ failure.

**Icosahedral symmetry:** An icosahedron is a geometric shape with 20 sides, each composed of an equilateral triangle.

**IgY:** An evolutionary ancestor of mammalian IgG found in the circulation of aves, reptiles, and amphibians, and is considered to be the functional homolog of mammalian IgG.

**Original antigenic sin:** Alteration in adaptive immune response (Both T and B cells), when preexisting memory cells of the previous infection encountered a heterotypic antigen. Such immune response is often reduced in degranulation, but increase in pro-inflammatory cytokine production, rendering such immune response ineffective in controlling viral infection, but more prone to cytokine storm.

**Pathogen-associated molecular patterns:** PAMPs are small molecular motifs that are shared between classes of microorganisms, and are recognized by PRRs in host cells to activate innate immune responses.

**Pattern recognition receptors:** PRR refers to an array of germline-encoded molecules sensing molecular motifs (PAMPs) conserved among different classes of microbes, and also recognize endogenous molecules called alarmins (DAMPs), released from injured host cells.

**Pyroptosis:** It is a form of cell death that follows pro-inflammatory signaling especially in inflammatory cells such as macrophages triggered by microbial pathogens.

**Tetravalent vaccine:** A tetravalent CYD vaccine contains live-attenuated DENV serotypes expressing the structural genes (encoding the membrane and envelope proteins) of each of the four DENV serotypes together with the genetic backbone of an attenuated 17D yellow fever virus (YFV) strain.

**Virion:** A single infectious virus particle that interacts with the host cells.
